# A single plasmid based CRISPR interference in *Synechocystis* 6803 – A proof of concept

**DOI:** 10.1371/journal.pone.0225375

**Published:** 2019-11-26

**Authors:** Prithwiraj Kirtania, Barbara Hódi, Ivy Mallick, István Zoltan Vass, Tamás Fehér, Imre Vass, Peter B. Kós

**Affiliations:** 1 Institute of Plant Biology, Biological Research Centre, Hungarian Academy of Sciences, Szeged, Hungary; 2 Doctoral School of Biology, University of Szeged, Szeged, Hungary; 3 Institute of Biochemistry, Biological Research Centre, Hungarian Academy of Sciences, Szeged, Hungary; 4 Department of Biotechnology, Faculty of Science and Informatics, University of Szeged, Szeged, Hungary; University of Hyderabad School of Life Sciences, INDIA

## Abstract

We developed a simple method to apply CRISPR interference by modifying an existing plasmid pCRISPathBrick containing the native *S*. *pyogenes* CRISPR assembly for *Synechocystis* PCC6803 and named it pCRPB1010. The technique presented here using deadCas9 is easier to implement for gene silencing in *Synechocystis* PCC6803 than other existing techniques as it circumvents the genome integration and segregation steps thereby significantly shortens the construction of the mutant strains. We executed CRISPR interference against well characterized photosynthetic genes to get a clear phenotype to validate the potential of pCRPB1010 and presented the work as a “proof of concept”. Targeting the non-template strand of *psbO* gene resulted in decreased amount of PsbO and 50% decrease in oxygen evolution rate. Targeting the template strand of *psbA2* and *psbA3* genes encoding the D1 subunit of photosystem II (PSII) using a single spacer against the common sequence span of the two genes, resulted in full inhibition of both genes, complete abolition of D1 protein synthesis, complete loss of oxygen evolution as well as photoautotrophic growth arrest. This is the first report of a single plasmid based, completely lesion free and episomal expression and execution of CRISPR interference in *Synechocystis* PCC6803.

## Introduction

CRISPR (*c*lustered *r*egularly interspaced *s*hort *p*alindromic *r*epeats)-Cas9 (*C*RISPR *a*ssociated *p*rotein 9) has revolutionized genome editing due to its simple execution and precision. The system was discovered in *Streptococcus pyogenes* and has primarily three working components: The ‘Cas9’ protein which has a single strand nuclease activity, a ‘tracrRNA’ complementary to the palindromic repeat sequences in the crRNA, which also forms RNA loop for Cas9 binding, and ‘crRNA’, which harbors palindromic repeats separated by ‘spacer’ sequences complementary to the targeted DNA sequence [1]. These three components are transcribed from a constitutive bi-directional promoter. Once transcribed, these two RNAs form a double stranded RNA with an RNA loop for Cas9 binding, which is then processed by cellular RNaseIII resulting in activated Cas9, which could screen the target genome and search for protospacer adjacent motif (PAM) sequences. Once such sequences are found lying next to those complementary to the crRNA, Cas9 will exert its nuclease activity [1]. This tri-component system was further simplified to a bi-component system where the tracrRNA and crRNA were combined as a single effector RNA named gRNA and Cas9 was placed under inducible promoters to control its expression for genome editing [2]. This system is widely adapted for both prokaryotic and eukaryotic gene editing [3].

An inactivated Cas9, termed as deadCas9 (dCas9), was developed which preserves the specific binding capacity to DNA targets of Cas9, with a complete loss of its nuclease activity [2]. This enzyme was able to perform transcriptional repression in bacteria in a process termed as CRISPR interference (CRISPRi) [4], which was then extensively characterized in *E*. *coli* [2, 3, 5] and being used for wide variety of purposes in genetic engineering and synthetic biology [6]. The basic working principle of this system’s application for transcriptional inactivation is that dCas9 will scan the genome until it finds a PAM, and if further pairing occurs between the crRNA and genomic DNA, it will bind strongly to the DNA, thereby preventing the transcription of the gene by hindering the binding and/or movement of RNA polymerase [3]. As there is no Dicer enzyme present in bacteria this RNA interference system swiftly gained high popularity [4, 7].

CRISPRi was further developed into two different systems in *E*. *coli*. ‘System one’ was developed by using the two component dCas9 and gRNA, which was shown to be highly versatile both in repressing the target genes and in tuning the CRISPRi system [3]. This system was shown to work from two plasmids containing gRNA and dCas9, respectively, that need to be transformed into the target bacterial cells. Alternatively, the gRNA and dCas9 cassettes can be integrated into bacterial genomes [8]. ‘System two’ of dCas9 relied on the original three component system of *S*. *pyogenes* CRISPR arrangement. The whole system could be executed in *E*. *coli* from a single plasmid by using the native *S*. *pyogenes* bi-directional promoter. This system was used as repressor and also as a transcriptional activator in *E*. *coli* [5]. The system was further developed by introducing a BsaI restriction site to facilitate easy cloning of multiple spacers. The resulting plasmid was called as pCRISPathBrick and was shown to be capable of multiplex repression of genes in *E*. *coli* [9]. The same system was also used to improve *E*. *coli* strain genome stability by silencing multiple insertion elements [10].

*Synechocystis* PCC6803 is an important model organism in scientific research. It was the first phototrophic organism to be fully sequenced offering an opportunity to study photosynthesis in unprecedented detail. It combines features from plants with features from microorganisms in scientifically attractive proportions: it holds a photosynthetic apparatus while being uncomplicated (simple, fast, versatile) to culture and to genetically transform. These and the available genome data are the main reasons to use *Synechocystis* PCC6803 in photosynthetic research. Accordingly, since its sequencing in 1996 about 160 papers are published annually with *Synechocystis* PCC6803 acting as the phototrophic model organism. The high number of studies with the same model organism facilitates meta-analysis of scientific data, it renders reproducibility across research groups more reliable and aids the standardization of specific scientific methods. Moreover, *Synechocystis* PCC6803 is also getting more and more attention in biotechnological applications [11], as well as in studies in systems biology [12]. However, because of the high and dynamically changing ploidy of cyanobacterial genome, this organism is a complex system to work with [13]. Accordingly, genome modification in *Synechocystis* PCC6803 requires a tedious process involving several steps of re-cultivation for complete segregation of the mutated chromosome. A number of attempts were made to edit cyanobacterial genomes using the CRISPR-Cas9 [14–16] and Cpf1 (also called Cas12) [17] genome editing machinery. However there were reports about toxicity of Cas9 proteins in cyanobacteria, which needs be avoided using tight regulation of Cas9 expression [18]. On the contrary, dCas9 does not affect cyanobacterial growth and it has been shown to allow the suppression of multiple genes, resulting in phenotypic changes in *Synechocystis* PCC6803 [8, 19, 20]. These studies were carried out using dCas9 in the above mentioned ‘system one’ methodology, in which the dCas9 and gRNA were integrated into the genome. This very step of generating the mutant is similar to the generation of knockout mutants using classical homologous recombination and further selection of mutants using chromosomal segregation [21], which is again a long process in itself to execute. Further, the resulting *Synechocystis* PCC6803 genome does not remain lesion free, rather it becomes an insertional mutant.

Here we developed a simple, fast and single plasmid-based strategy of CRISPR-dCas9 system following the above mentioned ‘system two’ approach. The resulting shuttle plasmid was created by replacing the original p15 origin of replication of pCRISPathBrick with a ‘conjugating origin of replication’ derived from RSF1010 and was named pCRPB1010. In this work, we demonstrate the functionality of this plasmid as “proof of concept” by showing suppression of three well characterized genes in *Synechocystis* PCC6803.

Through the episomal expression of CRISPR-dCas9, we were able to achieve significant repression of *psbO* and *psbA (psbA2 and psbA3)* genes that code for the manganese-stabilizing protein and for D1 reaction center subunit of PSII, respectively_._ The rescue of the mutants was fast and straightforward as it did not necessitate chromosomal segregation step that is characteristic in the other methodology. The phenotypic changes resulting from the gene suppressions were in good agreement with earlier studies [22, 23]. With this work we report for the first time a simple method to repress multiple genes using a single plasmid containing dCas9 in *Synechocystis* PCC6803, and demonstrate the potential of the technique to be applied in further studies requiring gene silencing.

It is noteworthy that last year a similar system has been successfully developed in *Nostoc* sp. PCC 7120 (*Anabaena* sp. PCC 7120) [24], a filamentous cyanobacterium, the most prominent model organism of cyanobacterial cell differentiation and nitrogen fixation. While here we present a system consisting of the original *S*. *pyogenes* genes and the native bidirectional promoter, they used plasmid-based dCas9-dependent silencing using a single guide RNA with engineered promoters and optimized ribosome binding site.

## Materials and methods

### Bacterial cultures and media

*Synechocystis* PCC6803 was used as the wild type strain (from here: *Synechocystis*). Cells kept under identical conditions in light have identical chlorophyll (Chl) to protein ratio and also identical cellular chlorophyll content even in psbO inactivated mutants [25]. Therefore throughout the present work the cultures were normalized to identical chlorophyll concentration as we found it more reliable [26] and less prone to artefacts (e.g. precipitates in the medium, differences in cell shape, interfering chemicals) than light scattering and Bradford method, respectively. Nevertheless, the growth of the cultures was monitored by reading light scattering (OD_720_) for simplicity. The cells were grown in BG11 medium under 40 μmol photons m^-2^ s^-1^ white light intensity, 3% CO_2_ enriched atmosphere, in 30°C. For maintaining the pCRPB1010-containing strains, BG11 was supplemented with chloramphenicol (25 μg mL^-1^). Solidified BG-11 agar plates were supplemented with 5 mM glucose when needed and the antibiotic concentrations and other conditions were kept as above. For experiments with WT and *psbO* repressed cells, *Synechocystis* cultures were grown to mid-log phase (OD_720_ 0.8) without antibiotics for two days and measurements were performed from cell suspensions adjusted to 5μg Chl mL^-1^. *psbA* repressed cells were maintained in respective antibiotics and 5 mM glucose. *Escherichia coli* DH5α was grown in Luria Broth supplemented with chloramphenicol (25 μg mL^-1^) for the cells harboring pCRPB1010 and with kanamycin (50 μg mL^-1^) for the cells containing pRK2013 [27].

### Plasmids used in this study

Plasmid pdCas9 was a gift of Luciano Marraffini (Addgene plasmid # 46569). It contains the dCas9 ORF, a tracrRNA and a crRNA resembling the native *S*. *pyogenes* organization under the control of a constitutive bi-directional promoter [5]. The spacer array of pdCas9 was modified to enable directional cloning of multiple spacers using the BsaI restriction endonuclease, as described previously [9]. The resulting plasmid, pCRISPathBrick has been characterized elsewhere to be able to suppress multiple genes in *E*. *coli* successfully [9, 10]. These plasmids contain the p15A origin of replication and are therefore unable to replicate outside *E*. *coli*.

**Fig 1:**
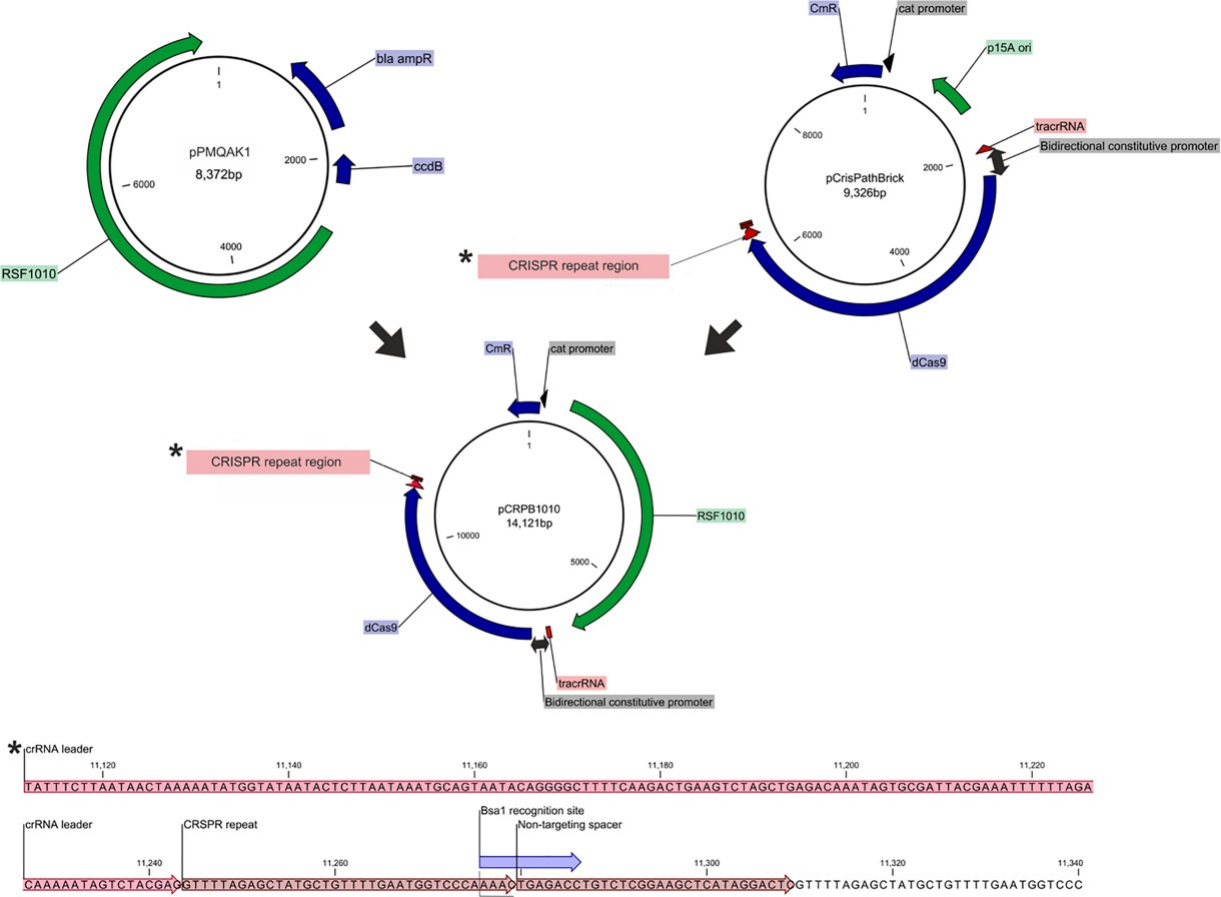
Schematic representation of pCRPB1010 development by combining regions from pCRISPathBrick and pPMQAK1. The CRISPR repeat region with nucleotide sequence of the linker part to which the target DNA segment should be cloned is shown in details. BsaI site is be also shown in the sequence.

pPMQAK1 was a kind gift of Peter Lindblad. This is a shuttle vector [28] that has pRSF1010 origin of replication, which enables its transfer to cyanobacteria through conjugation. We amplified this region by PCR with respective restriction cut sites (see Table 1) and by replacing the p15 oriC in pCRISPathBrick we created pCRPB1010 (Fig 1). The spacers were designed using CLC-main workbench following the instructions published earlier [9], and ordered as complementary oligonucleotides of 66 bp each. The oligonucleotides were phosphorylated, annealed and then cloned into pCRPB1010 using BsaI-restricted plasmids, transformed into *E*. *coli* DH5α and selected on antibiotic plate. The clones were confirmed by PCR using the corresponding primers (Table 1). The PCR fragments were confirmed by restriction fragment lengths using multi-cutter restriction endonucleases. The resulting plasmid pCRPB1010 was 14.5 kb in size and was not possible to naturally transform into the *Synechocystis* cells. Therefore a bi-parental mating between *E*.*coli* containing pRK2013 (helper plasmid) and pCRPB1010 and *Synechocystis* PCC6803 was performed [29], relying on the conjugative origin of replication RSF1010, as follows.

**Table 1:** List of oligonucleotides and primers used for this study.

Oligonucleotide name	Sequence
**psbO_sp1_fw**	AAACAAACCATGAGGTTTCGTCCGTCTATTGTGGGTTTTAGAGCTATGCTGTTTTGAATGGTCCCA
**psbO_sp1_rev**	GTTTTGGGACCATTCAAAACAGCATAGCTCTAAAACCCACAATAGACGGACGAAACCTCATGGTTT
**Position in *Synechocystis sp*. PCC 6803 genome: 2085922–2085951 negative, ORF_ID:sll0427 (T)**	
**psbO_sp2_fw**	AAACCGTTTTTATATAGCGGCAGTGCCTTTGCGGGTTTTAGAGCTATGCTGTTTTGAATGGTCCCA
**psbO_sp2_rev**	GTTTTGGGACCATTCAAAACAGCATAGCTCTAAAACCCGCAAAGGCACTGCCGCTATATAAAAACG
**Position in *Synechocystis sp*. PCC 6803 genome: 2085862–2085891 negative, ORF_ID:sll0427 (T)**	
**psbA23_sp_Fw**	AAACTCGCGCTGTTGGAGAGTCGTTGTCATTTGGGTTTTAGAGCTATGCTGTTTTGAATGGTCCCA
**psbA23_sp_rev**	GTTTTGGGACCATTCAAAACAGCATAGCTCTAAAACCCAAATGACAACGACTCTCCAACAGCGCGA
**Position in *Synechocystis sp*. PCC 6803 genome: 1818789–1818818 positive, ORF_ID:sll1867 (NT), 7225–7254 negative ORF_ID:slr1311 (NT)**	
**FP_RSF1010**	AACCGCGGAGTTCTTTTACCCTCAGCCG
**RP_RSF1010**	AAATCTAGAATGGTATTACCAATTAGCAGG
**pCRPRB_ins_FP**	GACAAATAGTGCGATTACGA
**pCRPRB_ins_RP**	AACGAGTCCTATGAGCTT
**dCas9_qPCR_FP**	CTCTATTCCCCATCAAATTC
**dCas9_qPCR_RP**	GCCAATGGACCAACATAA
**dCas9_PCR_FP**	CTCTATTCCCCATCAAATTC
**dCas9_PCR_RP**	TTCCTCAATCATCTCCCTATC
**psbA2_fw**	AGTCAGTTCCAATCTGAACATCG
**psbA2_rev**	TGACAAAACTGTTCCCACAAGG
**psbA3_fw**	ATACATAACCGGCTCCCAAGC
**psbA3_rev**	TGACAAAACTGTTCCCACAATGA
**psbO_fw**	GTTTCGTCCGTCTATTGT
**psbO_rev**	GGAAGAAATTTCAGGGCA

### Transformation of *Synechocystis* via biparental mating

The plasmids containing different spacers were chemically transformed into helper *E*. *coli* DH5α strain containing pRK2013. The cells containing dual plasmids were rescued on dual antibiotic plates (kanamycin and chloramphenicol). From overnight grown *E*. *coli* cells 0.1 mL culture was mixed with 1 mL of mid-log phase *Synechocystis* PCC6803. After centrifugation at 4500 rpm for 10 min at room temperature, 1 mL supernatant was discarded. The pellet was re-suspended into remaining medium and spotted on a BG11-containing agar plate with 5% LB. After two days the spot was scraped and spread on BG11 agar plates with chloramphenicol (25 μg mL^-1^) and glucose (5 mM). After 5 to 7 days a green lawn appeared. The lawn was scraped into 20 mL BG11 medium containing chloramphenicol (and 5 mM glucose in case of targeting *psbA* genes). After 3 days the culture became dense and from the liquid culture dilution plating was done on selective medium to isolate a single *Synechocystis* colony. In another 5-days, single colonies appeared on the plate. One colony was inoculated into chloramphenicol-containing (25 μg mL^-1^) liquid broth and further maintained in liquid culture and also stored at -80°C in the presence of 7.5% DMSO.

### Designing spacer sequences

Spacers were designed according to the published protocol [9], using *Synechocystis* PCC6803 genomic sequence (GCA_000009725.1) (see Table 1). For *psbO*, there were two spacers targeting the non-template DNA strand inside the coding region. Spacer1 and spacer2 were designed to target sequences starting at 5 bp upstream and 60 bp downstream of the start codon, respectively. For *psbA2* and *psbA3* a single spacer was designed targeting a 100% identical sequence span starting 4 bp upstream of each start codon of the template strand. We show the design of the targeted DNA sequences in the S1 Fig.

The spacers were synthesized by Eurofins Genomics.

### Biophysical measurements

Steady-state rates of oxygen evolution were measured at 30°C using a Hansatech DW2 oxygen electrode at a light intensity of 2000 μE m^-2^s^-1^in the presence of 0.5 mM 2,5-dimethy-p-benzo-quinone (DMBQ) as electron acceptor. 1 mL of cells at 5 μg Chl mL^-1^ was used in each measurement and the temperature of the culture was kept at 30°C by a circulator bath.

PSII activity was assessed by measuring the changes of variable Chl fluorescence values. The flash-induced Chl fluorescence measurements were performed with an FL 3000 Fluorimeter (Photon Systems Instruments Ltd.), using 1 mL samples. Measurements were repeated on three biological replicates at preset time points (see Fig 1). The data were visualized and evaluated using the Fluorwin software and Origin.

### Western blot

Cell suspensions were adjusted to 5 μg Chl mL^-1^. 2 mL aliquots of the suspensions were pelleted and kept at -20°C for further processing. The cell pellets were dissolved in 3X SDS-PAGE loading buffer. The 6M urea-PAGE was run following the published protocol for cyanobacteria [30]. Immunoblotting was carried out by transferring the proteins on to nitrocellulose membrane for 3 h in a cold-room at 25 V. The membrane was blocked using 10% skimmed milk in TBS. Primary antibodies against PsbO and PsbA (D1) were obtained from Agrisera AB, Sweden. Primary antibodies raised in rabbit were added to skimmed milk at a dilution of 1:2000 and incubated for 2 hr at room temperature. Goat-anti-rabbit-ALPO secondary antibody conjugate was used in 1:5000 dilution and visualized using NBT-BCIP, scanned, and analyzed using Image J software to calculate the band intensity.

### Quantitative PCR

15 mL cell suspension was added to 15 mL of ice-cold ethanol containing 5% phenol, mixed immediately and centrifuged at 10000 g for 10 min at 4°C. The supernatant was discarded and the pellet was resuspended in 0.5 mL of resuspension buffer (0.3 M sucrose, 10 mM NaOAc pH 4.5) and centrifuged again, resuspended in 0.25 mL of resuspension buffer and 37.5 μL of 0.5M EDTA was added. The cells were exposed to 4 freeze-thaw cycles and collected by centrifugation as above. Total RNA was isolated from the harvested cells using Direct-Zol RNA miniprep kit (Zymo Reseach). 500 ng of RNA was reverse transcribed using High-Capacity cDNA Reverse Transcription Kit (ThermoFisher) and the residual DNA was removed using TURBO DNA-free™ Kit (Invitrogen). cDNA corresponding to 10 ng RNA was used as template in the subsequent qPCR reactions. The primers used are listed in the primer list (Table 1).

### Assessment of growth rate

Cells were grown at 30±C under the conditions described above in the presence of respective antibiotics to an approximate OD_720_ of 2.0. For *psbA* repressed cells 5 μg glucose was also supplied. The cell cultures were then diluted to OD_720_ 0.2 and kept under the same conditions and the OD_720_ was measured every 24 hr.

### Statistical analysis

Where applicable, mean values and standard deviations are shown. The significance of statistical differences was tested using t-test in GraphPad Prism software at p≤0.05 significance level.

## Results and discussion

Metabolic engineering as well as exploration of protein functions and metabolic pathways often necessitates the generation of mutants deficient in certain genes or gene sets. Due to the polyploidy of the cells, creating deletion mutants in *Synechocystis* PCC6803 (from here: *Synechocystis*) is always a tedious and time-consuming process. Moreover, classical methods of creating mutants using homologous recombination have the limitation of altering one locus at a time. CRISPR-Cas9 has revolutionized genome editing and opens up the possibility to knock out multiple genes in a single shot. Earlier studies indicated Cas9 toxicity in cyanobacteria [18], which limits its utility in *Synechocystis*. On the other hand, a nuclease-inactivated Cas9 protein named dCas9 has no toxicity issues and CRISPR interference for transcriptional inactivation using this protein has been successfully implemented to downregulate multiple genes in *Synechocystis* [8, 19, 31]. However, this process was not lesion free to the genome and initial selection was tedious as it involves homologous recombination.

### Creating pCRPB1010

pCrisPathBrick was a single plasmid developed to induce CRISPR interference in *E*. *coli* using an inactivated Cas9 called dead Cas9 (dCas9), which allows cloning multiple spacers and thereby results in multiplex gene repression in *E*. *coli* [9]. Since the p15A origin of replication in this vector does not function in cyanobacteria, we replaced it with the replication origin of a broad-host range plasmid pRSF1010 that can replicate in various cyanobacterial strains [32]. This promoter was amplified from pPMQAK1, an *E*. *coli–Synechocystis* shuttle vector [28] (Fig 1). The resulting recombinant conjugative plasmid pCRPB1010 can therefore replicate both in *E*. *coli* and *Synechocystis* with all necessary parts of the dCas9 machinery. This allowed us to test this plasmid for transcriptional inactivation of genes in *Synechocystis*. We have also optimized the original conjugation method [32]. The plasmid was transferred into *Synechocystis* by bi-parental mating using a mutant *E*. *coli* containing the helper plasmid pRK2013 [27]. The technique developed here circumvents the above mentioned issues and demonstrates (discussed below) that multiple genes can be downregulated by using a CRISPR interference system with episomal expression from a single plasmid in *Synechocystis*. The time needed for creating a mutant strain using this technique is about 20 working days (Table 2).

**Table 2:** Timeline of applying CRISPR interference using pCRPB1010.

Day 0	Oligo (spacers) annealing, digestion of the vector (pCRPB1010), ligation overnight at 4±C.
**Day 1**	Transformation of *E*. *coli* (DH5-α).
**Day 2**	Single colony screening with respective antibiotics.
**Day 3**	PCR screen, using 2 μl of liquid culture to ensure spacers. Isolation of the desired plasmids from positive clones.
**Day 4/5**	Chemical transfer of the desired plasmids into helper *E*. *coli*. PCR screen to confirm the insert.
**Day 6**	Overnight growth of positive helper *E*. *coli* in 2 ml LB medium with antibiotics.
**Day 7**	Conjugation of helper *E*. *coli* with the wild type *Synechocystis sp*. PCC 6803.
**Day 9**	Selection plating of the *E*. *coli* and *Synechocystis sp*. PCC 6803 conjugation mix onto BG-11 agar plates with antibiotics.
**Day 14/15**	Transfer of *Synechocystis sp*. PCC 6803 single colonies into BG-11 liquid media.
**Day 20**	PCR screen of *Synechocystis sp*. PCC 6803 for the presence of pCRPB1010.

### Targeting the *psbO*, *psbA2* and *psbA3* genes

**Fig 2:**
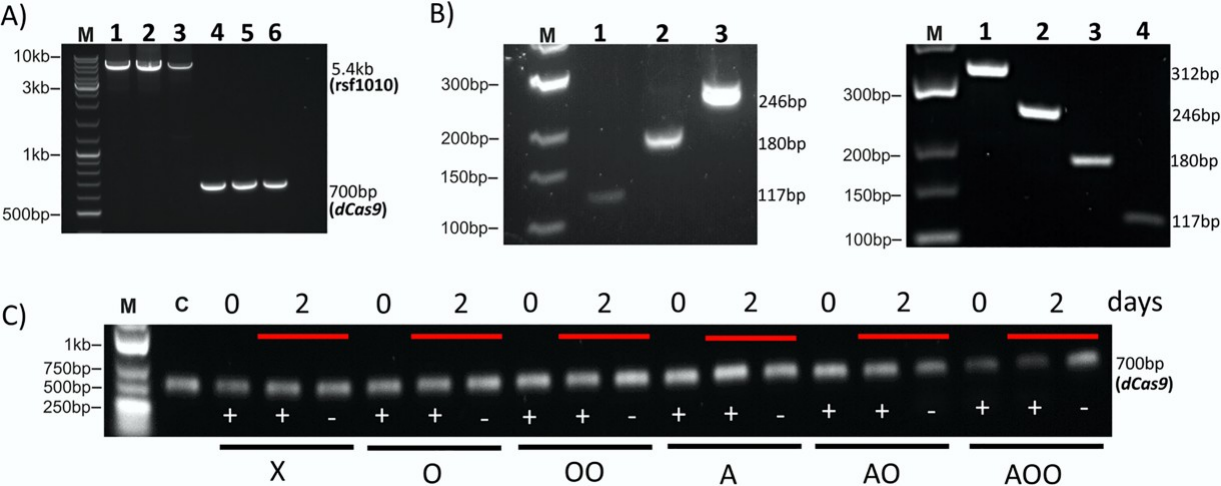
Validation of the pCRPB1010 plasmid and spacers. (A) PCR analysis assessing the presence of pCRPB1010 in *Synechocystis* after conjugation. RSF1010 region (lanes 1 to 3) and *dcas9* gene (lanes 4 to 6) are confirmed in the strain with the empty plasmid (pCRPB1010, lanes 1 and 4), single spacer (pCRPB1010+psbO, lanes 2 and 5) and double spacer (pCRPB1010+*psbO*+*psbA*, lanes 3 and 6). (B) Validation of spacers in pCRPB1010 by PCR amplicon length. Left panel: *psbO* silencing: pCRPB1010 (no spacer, lane 1) pCRPB1010-psbO (single spacer, lane 2) and pCRPB1010–psbO (DS) (double spacer, lane 3). Right panel: *psbO*, *psbA2 and psbA3* silencing: pCRPB1010–psbO(DS)–*psbA* (triple spacer, lane 1); pCRPB1010–*psbO*–*psbA* (double spacer, lane 2); pCRPB1010–*psbA* (single spacer, lane 3); pCRPB1010 (no spacer, lane 4). (C) Assessment of plasmid stability in the cells. The presence of the plasmid was verified using PCR after keeping the cultures with no antibiotics for two days for photosynthetic activity measurements. The notation of the strains are as follows: X, “empty” plasmid with no insert; O and OO: single and double spacers targeting *psbO*; A: spacer targeting *psbA*. Lane M is molecular weight marker, lane C is the amplicon using the purified plasmid DNA, as positive control. The presence (+) and absence (-) of the antibiotic is shown under the bands.

After transferring pCRPB1010 into *Synechocystis*, the presence of pCRPB1010 inside the cell was confirmed by colony PCR using primers designed for RSF1010 and dCas9, respectively, resulting in 5.4 kb and 700 bp bands (Fig 2A). The “empty plasmid” does not differ in this respect from the ones with spacers.

The expression of dCas9 was validated using quantitative PCR, using *trpA* as internal control. The results show that (1) dCas9 is expressed in all mutant strains (including the one with empty plasmid) and (2) the expression of dCas9 (C_T_ = 26,2 ± 0,19) is as stable as the housekeeping gene (C_T_ = 24,5 ± 0,24) across the strains and the expression level is comparable to moderately expressed genes (Table 3). The expression of dCas9 proved that the native bidirectional constitutive promoter of *S*. *pyogenes* between the genes of the tracrRNA and dCas9 is active in *Synechocystis*, as this promoter controls the expression of the whole system.

**Table 3:** Assessment of dCAS9 gene expression in the strains under study. qPCR data are represented as cycle numbers. Higher number represents lower expression. All “no template controls” and “no RT controls” showed no amplification and were omitted from the table. WT: Wild type; X: plasmid with no spacer. The spacers in the plasmids are as follows. O and OO: single and double spacers against *psbO*; A: spacer against *psbA*.

	WT	X	O	OO	A	AO	AOO
dCAS9	-	26,0	25,9	26,4	26,3	26,2	26,2
trpA	24,5	24,6	24,7	24,4	24,0	24,6	24,6

Next, spacers targeting *psbO* and *psbA* genes were designed (detailed in Experimental Procedures). The first targeted gene, *psbO* is present in the genome in one single copy with no paralogs. It is a well characterized non-essential photosynthetic gene, the deletion of which results in characteristic phenotype change [22]. The non-template strand of *psbO* was targeted and two spacers binding to two different segments inside the coding region were designed (see S1 Fig). Either one, or both of the two spacers were cloned into pCRPB1010 in order to test if the second spacer can have additional silencing effect.

As a second test we aimed to assess the capacity of the system for targeting multiple genes, and wanted to see if a single spacer can concomitantly silence multiple genes. To this end a single spacer targeting an identical segment of both *psbA2* and *psbA3* was designed. *psbA* genes code for D1 protein of the second photosystem, a protein of the highest turnover rate under most conditions. This gene has three paralogs in the *Synechocystis* genome, one of which (*psbA1*) has a somewhat divergent DNA sequence and is hardly expressed under normal conditions [33]. The two other copies (*psbA2* and *psbA3*) [34] code for identical protein sequences and their DNA sequence similarity is so high that it was possible to design a common spacer targeting both of them (see S1 Fig).

Contrary to the spacers targeting *psbO*, we targeted the template strand of *psbA* genes to understand if there is any difference between the two approaches, as there are contradictory reports in the literature about targeting a particular DNA strand to achieve higher transcriptional repression [35].

This *psbA*-specific spacer alone and with single and double *psbO*-specific spacers, was cloned into pCRPB1010. The latter two constructs were created to validate multiple gene targeting capacity of this plasmid. The insert lengths were confirmed using PCR following the protocol published earlier [9] (Fig 2B). Since biophysical measurements (see below) were carried out using cells kept without antibiotics for two days, the presence of the *dCas9* gene (and hence the plasmid) was checked from cultures grown similarly. The plasmid remained stable without antibiotics inside the *Synechocystis* for two days (Fig 2C). It is also noteworthy that the plasmid pCRPRB1010 in *Synechocystis* was still present in the cells kept in light even after a month of serial re-culturing without antibiotic.

### Effect of silencing on gene expression and on the amount of synthesized proteins

**Fig 3:**
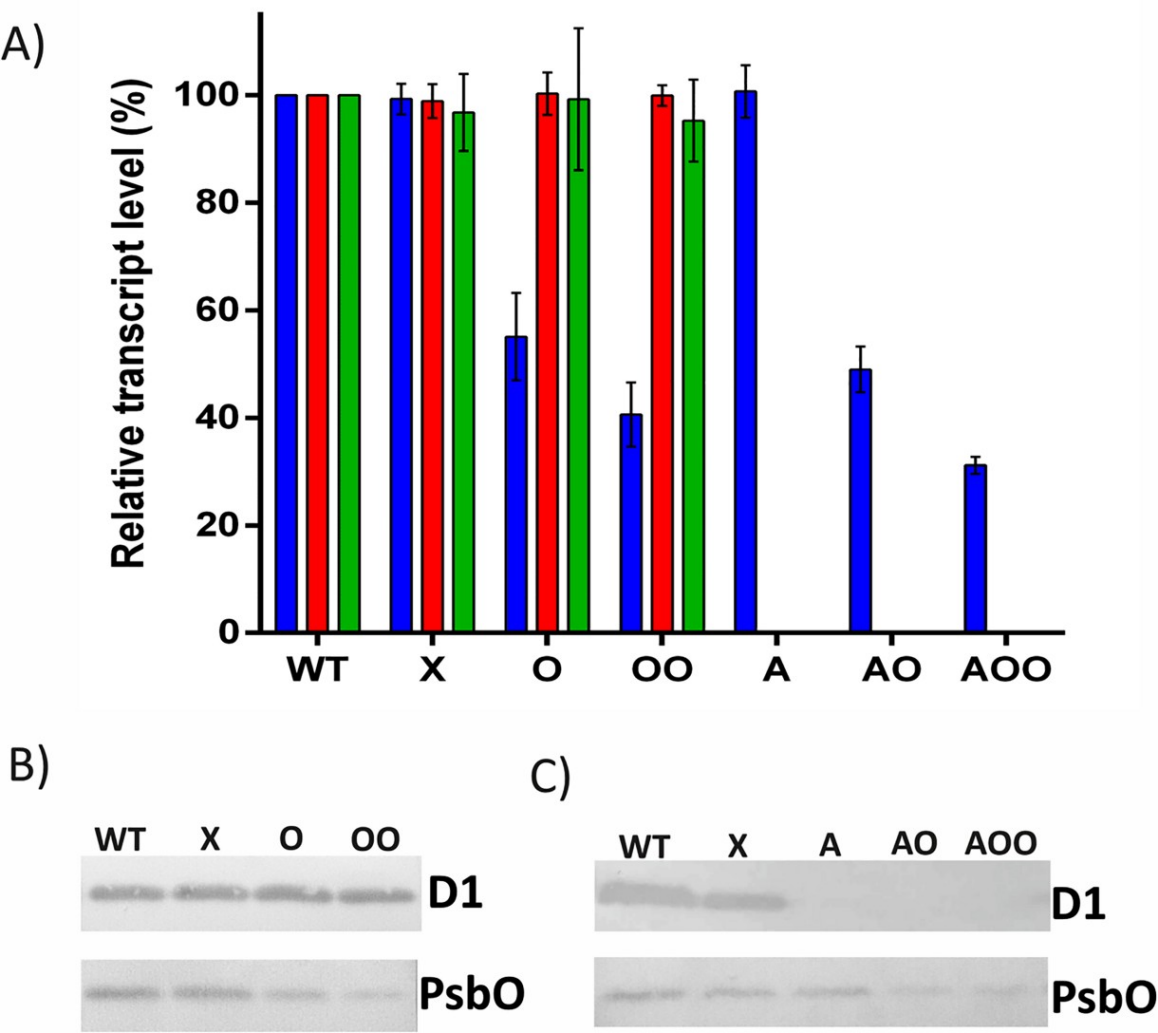
Assessment of the gene expression and the synthesized proteins. (A) *psbO*, *psbA2* and *psbA3* gene expression levels in various strains measured by qPCR. The expression levels were measured in three replicates and normalized to the wild type (WT) strains. The investigated genes are represented by blue bar (*psbO*), red bar (*psbA2*) and green bar (*psbA3*). The strains harbor pCRPB1010 (only plasmid with no spacers, X), single spacer (O) and double spacer (OO) targeting *psbO*, single spacer targeting *psbA* (A), combined single spacers targeting *psbA* and *psbO* (AO), single spacer targeting *psbA* and double spacer targeting *psbO* (AOO). (B) protein expression assessment using western blot in strains where *psbA* genes are not targeted. Strain designations are as above. D1 is used as internal control for verifying identical sample amounts. (C) protein expression assessment where both protein types are silenced. Strain designations are as above.

In order to see the effect of the dCas9 system on gene expression, cells from exponential growth phase were sampled and the expression of *psbO*, *psbA2* and *psbA3* were assessed using quantitative real-time PCR (qPCR), using *trpA* gene as an internal control. The expression levels were evaluated as the percent of the ones in the wild type strain (WT). Both, in the absence and presence of the *psbA*-targeting spacer, the single targeting of *psbO* (O, AO, respectively) resulted in very significant but not complete suppression of the expression level, and the double targeting of *psbO* gene (OO, AOO, respectively) showed significant improvement of the repression. On the contrary, the *psbA* targeting resulted in complete abolishment of the expression of both *psbA* genes (Fig 3A).

In order to see the effect of the dCas9 system on protein synthesis level, cells from exponential growth phase were sampled for western blot analysis. The cultures were adjusted to 5 μg/mL chlorophyll-a concentration and equal amounts were separated on SDS-PAGE. Western blot results showed a decrease in the intensity of bands in the presence of single and double spacers targeting *psbO* (Fig 3B). As an internal control, D1 protein was analyzed from the same samples and no change in expression was observed (Fig 3B).

When both *psbA* and *psbO* genes were targeted, western blot results showed that D1 band is absent in *psbA*-targeted strains (Fig 3C). In cells where only *psbA* was targeted, the PsbO amount was unchanged, whereas in combined targeting cases single and double spacers against *psbO* resulted in decreased PsbO band intensity. The changes in band intensities were in good agreement with the gene expression levels of the corresponding strains.

### Phenotypic assessment of the silencing

**Fig 4:**
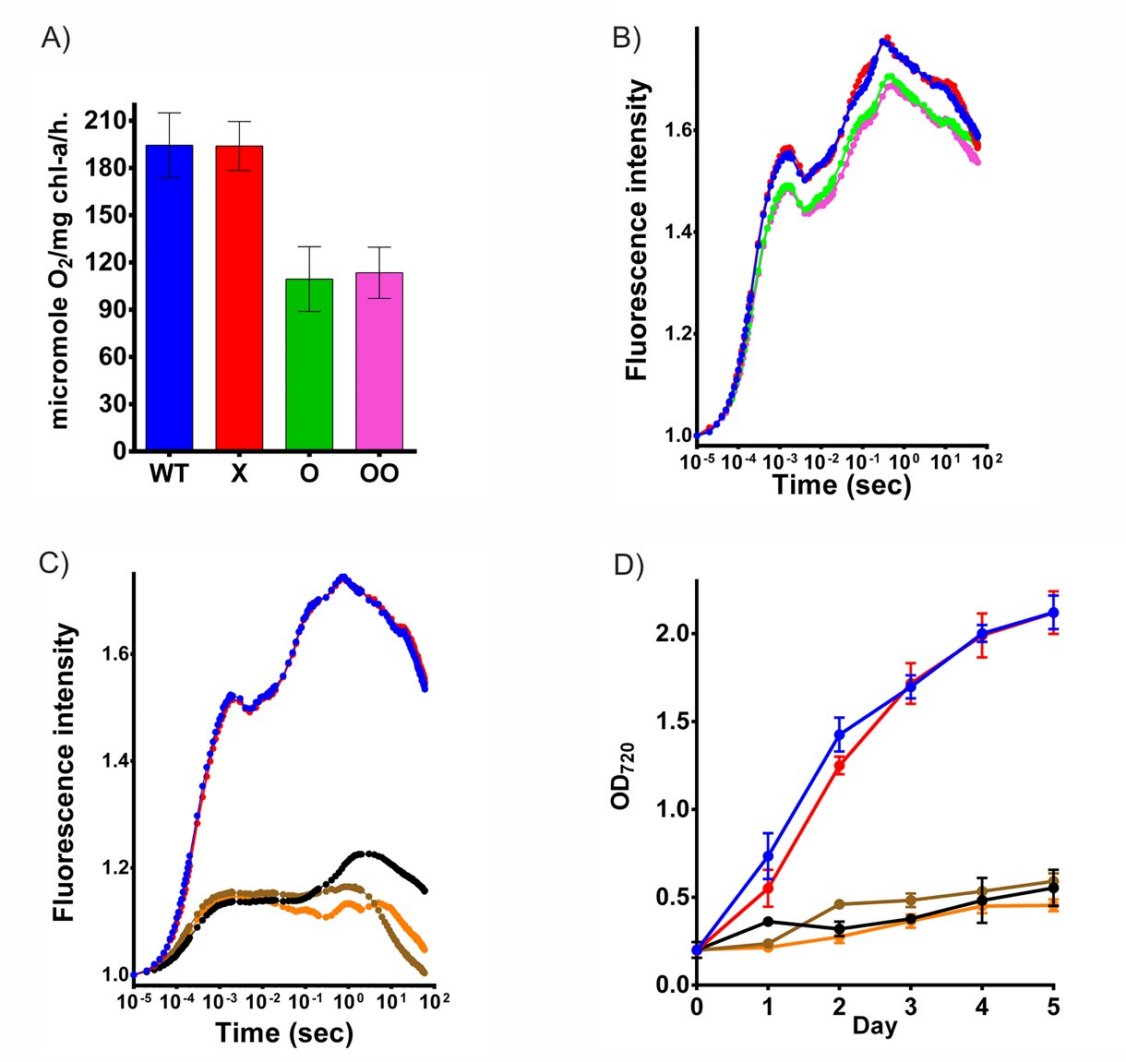
Phenotypic effects of gene silencing. (A) Oxygen evolution in different *Synechocystis* strains. Strain designations are as in (Fig 3). (B) Fast chlorophyll-a fluorescence induction curves of *psbO*-suppressed cells. Wild type *Synechocystis* strain (blue), *Synechocystis* strain containing plasmid with no spacer (red), *Synechocystis* strain with single spacer (green) and with double spacer (pink) for *psbO*. (C) Fast chlorophyll fluorescence induction curves of *psbO*- and *psbA*-suppressed cells. Wild type *Synechocystis* strain (blue), *Synechocystis* strain containing plasmid with no spacer (red), *Synechocystis* strain with spacer targeting *psbA* (black), with spacer targeting *psbA* and a single spacer against psbO (brown) and with spacer targeting *psbA* and a double spacer targeting *psbO* (orange). (D) Growth curves of D1 suppressed *Synechocystis* strains compared to wild type. Color codes are as in panel C.

The effect of gene silencing on the photosynthetic activity was assessed via biophysical characterization of the cultures, using oxygen evolution rate and fast chlorophyll fluorescence measurements. These measurements were carried out in *Synechocystis* cultures balanced to 5 μg/mL chlorophyll-a concentration. PsbO is a manganese stabilizing protein characterized earlier, which has a role in molecular oxygen evolution in *Synechocystis* and other photosynthetic organisms. Complete disruption of *psbO* results in about 70% of reduction in oxygen evolution rate as reported earlier [22]. In concert with these data we found that the oxygen evolution rate of the cultures containing the plasmid with both a single and double spacer against *psbO* was decreased by about 40% as compared to the wild type strain (Fig 4A). In case of D1 deletion mutants, previous studies showed that disruption of D1 prevents the synthesis of PSII and leads to complete abolition of oxygen evolution and photosynthetic growth [23, 36, 37]. In concert with these data, no oxygen evolution could be observed in case of strains containing spacers targeting *psbA* genes.

In order to gain a better understanding on the PSII activity of the *psbO*- and *psbA*-suppressed cells, fast chlorophyll fluorescence induction measurements were performed. Variable chlorophyll fluorescence, i.e. the rise of the fluorescence yield from the initial F_o_ level to the maximal F_m_ level assesses electron transport capacity through PSII. The *psbO*-suppressed cells showed somewhat decreased variable fluorescence induction compared to wild type and pCRPB1010-containing cells (Fig 4B), which in concordance with the observed decrease in oxygen evolution rate, indicates some inhibition of PSII activity. In *psbA*-silenced cell cultures the fast fluorescence transients showed drastic changes (Fig 4C) in agreement with the absence of D1 protein and the completely ceased oxygen evolution.

In spite of the above shown decrease in photosynthetic activity of *psbO*-suppressed cultures, we did not observe significant difference between their growth rates. On the other hand, *psbA-*suppressed strains could only be maintained with 5 mM glucose added and did not grow in photoautotrophic conditions (Fig 4D), which was in agreement with previously published observations [37]. This result further corroborates the finding of the protein analysis above, showing the absence of D1 protein and hence complete inactivation of PSII caused by the activity of dCas9 expressed from a single plasmid inside *Synechocystis*.

Previous studies reported 50–95% repression of gene expression in *Synechocystis* using dCas9 [8, 19]. In those investigations, the genome integration strategy was used and the non-template strands of the genes were targeted. Here we tested the targeting of each strand. We found significant but not full repression of the targeted gene with non-template strand targeting, in agreement with the above publications. However, when the template strand was targeted, a complete loss of function was observed. Whether this complete inactivation of the photosynthetic apparatus was the consequence of the choice of the template strand targeting, is hard to tell at the current point without further investigation. Nevertheless, it is obvious that using this targeting method it is possible to achieve very strong repression in *Synechocystis* for complete loss of function of two genes using one single spacer.

In conclusion, the work presented here is the first report of an easy and single-plasmid based execution of CRISPR interference using dCas9 in *Synechocystis*, which is capable of concomitantly downregulating multiple genes in a lesion-free manner. This is a first report showing that the native CRISPR-array from *S*. *pyogenes* can also function well in *Synechocystis* with its fully functional bidirectional promoter between the genes of the tracrRNA and *dCas9* and also the first report where dCas9 was used to modulate the photosynthetic machinery in *Synechocystis*.

Our findings are in good agreement with the results of Higo et al. [24] in *Nostoc* sp. PCC7120. Taken together this technique will facilitate and speed up the molecular level investigations and genetic engineering of these organism as well as other cyanobacterial strains.

## Supporting information

S1 Fig Targeted genomic sequences of *psbO* and *psbA* genes. Sequences around the start codons of *psbO* (A) and the two aligned active *psbA* (B) genes. The targeted sequences (spacer sequences) are shown along with the corresponding PAM motifs. The first spacer binding region of *psbO* and the one of *psbA* overlaps with the start codon, but in different orientation.
(TIF)
